# A new tool for assessing short debriefings after immersive simulation: validity of the SHORT scale

**DOI:** 10.1186/s12909-019-1503-4

**Published:** 2019-03-12

**Authors:** Etienne Rivière, Etienne Aubin, Samuel-Lessard Tremblay, Gilles Lortie, Gilles Chiniara

**Affiliations:** 1 Department of Internal Medicine, Haut-Leveque Hospital, University Hospital Centre of Bordeaux, Pessac, France; 2 Medical Faculty, Bordeaux University, Bordeaux, France; 3 SimBA-S Simulation Centre, University and Hospital of Bordeaux, Quebec, Canada; 4 Apprentiss Centre (simulation centre), Laval University, Quebec, Canada; 5 Independent Researcher, Quebec, Canada; 6 University Institute of cardiology and pneumology of Quebec, Quebec, Canada; 7 Emergency Unit, Levis Hotel-Dieu Hospital, University Hospital of Quebec, Lévis, Canada; 8 Department of Anesthesiology and Intensive Care, Laval University, Quebec, Canada

**Keywords:** Simulation training, High-fidelity simulation training, Patient simulation, Educational measurement, Formative feedback, Generalizability theory, SHORT

## Abstract

**Background:**

Simulation is being increasingly used worldwide in healthcare education. However, it is costly both in terms of finances and human resources. As a consequence, several institutions have designed programs offering several short immersive simulation sessions, each followed by short debriefings. Although debriefing is recommended, no tool exists to assess appropriateness of short debriefings after such simulation sessions. We have developed the Simulation in Healthcare retrOaction Rating Tool (SHORT) to assess short debriefings, and provide some validity evidence for its use.

**Methods:**

We designed this scale based on our experience and previously published instruments, and tested it by assessing short debriefings of simulation sessions offered to emergency medicine residents at Laval University (Canada) from 2015 to 2016. Analysis of its reliability and validity was done using Standards for educational and psychological testing. Generalizability theory was used for testing internal structure evidence for validity.

**Results:**

Two raters independently assessed 22 filmed short debriefings. Mean debriefing length was 10:35 (min 7:21; max 14:32). Calculated generalizability (reliability) coefficients are φ = 0.80 and φ-λ3 = 0.82. The generalizability coefficient for a single rater assessing three debriefings is φ = 0.84.

**Conclusions:**

The G study shows a high generalizability coefficient (φ ≥ 0.80), which demonstrates a high reliability. The response process evidence for validity provides evidence that no errors were associated with using the instrument. Further studies should be done to demonstrate validity of the English version of the instrument and to validate its use by novice raters trained in the use of the SHORT.

**Electronic supplementary material:**

The online version of this article (10.1186/s12909-019-1503-4) contains supplementary material, which is available to authorized users.

## Background

Simulation-based education has proven its efficacy in healthcare and brought clinical benefits to patient care [1, 2]. For these reasons, it is being increasingly used worldwide. As an educational method, it includes several modalities. Among those, immersive simulation aims to reproduce an authentic clinical experience for learners (the so-called “high-fidelity simulation”), with an expert debriefing congruent to learners’ needs. While very effective, this modality is also very costly, both in material and human resources. This constraint, together with the high number of students in the different healthcare fields, is a strong impetus for optimizing time allotted to immersive simulation. Consequently, many institutions propose shortened activities designed to fit a higher number of students and scenarios in the time slots available.

According to best practices in simulation, an immersive simulation session must be followed by a systematic debriefing in order to be effective [3]. However, when little time is available, debriefing can be difficult. In a way, short debriefings are a peculiar type of debriefing that generate specific issues for the instructor, e.g. limited time for emotional release, reduced participant interaction, restricted probing of participants’ performance gap, limited time for discussing overall issues and overarching principles [4], and reduced time and opportunities to foster transfer of learning. The instructor usually focuses on one particular issue and uses consecutive simulated sessions and debriefings to build an “overall” feedback, addressing issues one after the other through successive short simulation sessions.

In addition to simulated sessions taking place in a simulation centre or in clinical units (in situ simulation), short debriefings could also be conducted after real clinical situations to improve team performance [5]. Assessing debriefing of real situations by a person trained in the use of debriefing assessment scales can provide a perfect learning opportunity for reflexive instructors to improve their performance. When simulation in healthcare is offered in a simulation center, assessing the quality of debriefings is a necessary component of a quality improvement program. As such, an instrument to evaluate short debriefings is needed. While other debriefing assessment instruments already exist, such as the Debriefing Assessment for Simulation in Healthcare (DASH) [6] and the Objective Structures Assessment of Debriefing (OSAD) [7], they do not address the specificities of short debriefings (10 min or less) addressed above. Inspired by DASH [6], and based on existing recommendations for debriefing [3] and on principles of learning transfer [8–11], we designed a rating scale called SHORT, for “**S**imulation in **H**ealthcare retr**O**action **R**ating **T**ool”.

This article describes the development process for the SHORT, and the validity evidence we collected to support its use, using the framework provided by the *Standards for educational and psychological testing* [12]. We also provide the psychometric data we measured to document its reliability, based on Generalizability Theory (GT) [13, 14].

## Methods

### Context

All debriefing videos used to assess the validity of the SHORT were recorded between August 2015 and April 2016, during Laval University’s regular simulation activities. Participants in the simulation sessions were PGY-1 and PGY-2 residents in emergency and family medicine. Instructors (debriefers) were faculty staff from the emergency and family medicine program, with varying degrees of formal training in debriefing. All instructors and participants gave prior written consent to be recorded and assessed.

### Instrument structure and content

The SHORT is a global rating scale that includes 5 items and a holistic expert assessment (*see the SHORT scale in the* Additional file 1). It is based on the cognitive principles for debriefing, as outlined by the CDR model [15]. The first five items are described as follows:

- “Environment”, describes the psychological ambiance in which debriefing takes place;
- “Debriefing structure” checks for the presence of an emotional phase during debriefing as well as learning summaries and the achievement of learning outcomes specific to the case;
- “Debriefing facilitation” assesses the way the debriefer facilitates debriefing by encouraging discussions, reorienting learners when they digressed from the objectives, and managing the potential resistant or disruptive learner;
- “Analysis” assesses how the debriefer considers the learners’ performance during the case and explores their cognitive frames (contextualizing)
- “Transfer” assesses how the debriefer highlights the overarching concepts and concrete strategies that are pertinent to managing the present case (decontextualizing) and a future similar case (recontextualizing).

Items are scored from 1 (harmful) to 5 (expert), using the specific cues provided under each score for each item. These cues are separated thematically by lines to facilitate accurate rating.

The expert holistic rating is independent of the 5 items and is rated on a Likert scale without specific cues, as follows:

- “1 = harmful”: undoes learning, or harms the credibility of the training or of the simulation modality;
- “2 = neutral”: learners gain no benefit from the debriefing, or the debriefer does not make the simulation modality relevant;
- “3 = must improve”: the debriefer encourages learning, but does not allow the simulation modality to be used at its optimal capacity;
- “4 = could improve”: the debriefer encourages learning significantly and allows the simulation modality to be used at its optimal capacity;
- “5 = expert”: the debriefer encourages learning significantly and could be cited as an example, or the debriefer could train other debriefers.

Detailed explanations are given in Additional file 1 *(the SHORT scale itself, and the rater’s guide to use the SHORT scale).*

### SHORT development

The SHORT is based on a French scale used at Centre Apprentiss (simulation center) (Additional file 2) at Laval University since 2010 for assessing debriefings of long immersive simulation activities, to improve debriefer competency and standardize practice. It was initially developed by GC based on a scale designed by Morgan Jaffrelot, MD, M(Ed) (Brest, France) and Georges Savoldelli, MD, M(Ed) (Geneva, Switzerland), itself inspired by the DASH rating scale. A preliminary French version adapted to short debriefings was developed by ER, SLT, GL and GC, and was tested by ER, SLT and GL on previously recorded short debriefings of simulation sessions. Iterative discussions led the authors to select the most important items to be assessed according to the literature and their own experience. Criteria guiding this iterative process were based on best practices for debriefings [3] and on the available literature on transfer of learning [16–21]. The following criteria were thus selected and developed: the need for an emotional phase, adequate context analysis and extraction of general principles from the situation, and systematic fostering of learning transfer. The tests allowed scale adjustment, and several iterative versions were developed and tested. The research being a part of program assessment, it was exempt from review by the institutional review board.

The SHORT instrument was then tested prospectively by reviewing video recordings of debriefing sessions. Two raters (ER & SLT) independently assessed the remaining short debriefings with the scale. Finally, the document explaining its use was conceived by all authors.

As the study was conducted with the French scale, it was translated in English by an English-native speaker, and then translated back into French by a different person to check the concordance between the original French version and the one generated by the first translation (double translation) [22].

### Demonstrations of validity

Using the *Standards for educational and psychological testing* as our framework, five categories of evidence are explored: test content, internal structure, relation to other variables, testing consequences and response process [23].

The **test content evidence for validity** evaluate whether the instructions and item content are relevant to the purpose of the tool. We described the tool elaboration, structure and content in the preceding section.

The **internal structure evidence for validity** could be summarized as the following questions. Are the relations between the items congruent with what is expected? Is the use of the SHORT generalizable to similar contexts? Both questions will be answered with Generalizability Theory (GT) [13, 14], using a G study. We will assess the differences between the items by analyzing their individual contribution to the total variance of the score, and their interactions. An absolute G coefficient (φ) will be used to assess the generalizability (reliability) of the results.

The **relation to other variables evidence for validity** evaluate whether the results correlate to other variables as expected. This source for validity evidence is beyond the scope of this study.

The **testing consequences evidence for validity** evaluate whether the conclusions drawn from the tool are correct. As the tool recommends training for scores of 3 or less, we’ll assess the reliability of those decisions with GT (Phi-lambda: φ-λ_3_). Also, since the SHORT should ideally be used by only one rater in short-staffed contexts, we will perform optimization studies (or D studies) to determine how many observations are needed to ensure reliability when only one rater assesses short debriefings.

The **response process evidence for validity** could be summarized by the following questions: do the raters use the tool appropriately? Are the raters familiar with the instrument? This will be considered in the discussion.

### Generalizability theory (GT)

GT has two components: a theoretical model and a mathematical model similar to ANOVA. It is interested in the generalization of an individual’s score to the average score of that person under all possible and acceptable conditions of the test. Through generalizability studies (G studies), it isolates the effects on the measure of specific sources of variance (“facets” or components), to identify those that introduce an error in measurement (a bias), by themselves and by interacting with other facets. It also provides measures of reliability (G coefficients). Finally, through optimization studies (D studies), GT can determine the optimal test conditions, i.e., the conditions that reduce errors and increase reliability.

Four facets are considered in the G study for the SHORT (Table 1). First are the de**b**riefers (B), which are the object of measurement, the one that has to be reliable and valid. The other three facets, which can introduce biases, are the **r**aters (R), the **i**tems of the scale (I), and the **o**bservations (O) of the short debriefings. All items are considered as invariant (*fixed facet* in GT terms), and the other components are considered as extracted from a very large target population (*infinite universe* in GT terms). The design of the G study is a mixed model “R x I x O:B”, meaning that all raters assess all the debriefers with all items (R x I x O), but the observations of the short debriefings are specific to debriefers: the O facet in *nested in* the B facet (O:B).

**Table 1:** Facets of the SHORT included for analysis

Facet	Number (*n*)	Universe size^a^	Face type	Description
Debriefer (B)	11	Infinite	Random	The object of measurement
Rater (R)	2	Infinite	Random	The raters or evaluators
Items (I)	6	6	Fixed	The items of the scale
Observations per debriefer (O:B)	2	Infinite	Random	The individual observations of a debriefing

^a^Universe size represents the universe of admissible values

As previously mentioned, the reliability of the scores is evaluated relative to the exact concordance of the scores (absolute G coefficient; φ), and according to the recommendation of training for scores of 3 or less (Phi-lambda: φ-λ_3_), i.e. by considering that all debriefers with a rating scale at 3 or below should undergo further debriefing training.

D studies determine how many observations will be needed to ensure reliability of the SHORT (i.e., reliability coefficient above 0.8) if only one debriefer assesses short debriefings. The value of 0.8 is chosen because it is an adequate level of reliability for high-stake assessments [24, 25]. Analyses were done with EduG 6.1-f (Educan, Longueil, Canada).

## Results

A total of 22 short debriefings were independently assessed by the two raters, each rater assessing 2 debriefing sessions for each of the 11 unique debriefers. The mean debriefing length was 10:35 (min 7:21; max 14:32). Once the scale was well assimilated by the raters, the time needed to complete an assessment after viewing the video was approximately 2 min, with a mean total assessment length of approximately 12 min. Each video was reviewed only once. Final ratings ranged from 1 (harmful) to 4 (could improve). The sources of variance in the SHORT scores are given in Table 2. Negative variances were judged negligible and thus were excluded from the last column. The calculated G coefficients are: φ = 0.80 and φ-λ_3_ = 0.82. If the holistic rating is removed from the analysis (i.e., only items 1 to 5 are included), the calculated G coefficients are: φ = 0.78 and φ-λ_3_ = 0.79. Calculating G coefficients for the holistic rating alone yields results of: φ = 0.78 and φ-λ_3_ = 0.82.

**Table 2:** Variance by source

Variance components	Sum of squares	*df*	Mean square	Corrected variance	%^a^
R	0.136	1	0.136	0.000	0.0
I	28.045	5	5.609	0.094	12.0
B	75.114	10	7.511	0.252	32.1
O:B	16.750	11	1.523	0.106	13.5
R × I	1.091	5	0.218	0.002	0.2
R × B	1.947	10	0.195	−0.005	0.0
R × O:B	2.750	11	0.250	0.042	5.3
I × B	29.205	50	0.584	0.102	13.0
I × O:B	10.250	55	0.186	0.000	0.0
R × I × B	8.826	50	0.177	−0.005	0.0
R × I × O:B, *e*^b^	10.250	55	0.186	0.186	23.8

^a^Proportion of corrected variance (i.e., percentage of total variance in the score introduced by each facet), when negative variances are rounded to 0

^b^This source of variance includes variance linked to the interaction of all the facets (R × I × O:B), as well as the unidentified residual variance (*e*). See text for further details

Table 3 shows the result of the D studies. It reports the generalizability (reliability) coefficient φ of the SHORT if it was used by only a single rater observing a variable number of debriefings by a given debriefer.

**Table 3:** G and D studies of the SHORT

Element	G Study	D study 1 (D1)	D study 2 (D2)	D study 3 (D3)
R	2	1	1	1
O:B	2	2	3	4
φ (0.00)	0.80	0.77	0.84	0.87
Error variance	0.063	0.074	0.049	0.037
Standard error of measurement	0.25	0.27	0.22	0.19

## Discussion

Using the *Standards for educational and psychological testing* [12] as the validity framework to assess the “SHORT” scale, four categories of evidence for validity were tested: test content, internal structure, testing consequences and response process.

The **test content** source of validity evidence was documented in the methods section. It provides an overview of the SHORT development process, which relies on previously published rating scales as well as theoretical foundations in simulation-based education and debriefing.

Several methods exist to assess the efficiency of a debriefing session. An easy way is to have learners fill a questionnaire after debriefing. However, this type of evaluation only assesses participants’ reactions, at the first level of the modified Kirkpatrick four-model evaluation pyramid (level 1: reaction) [26]. The use of a global rating scale to assess instructor’s efficiency in debriefing is more appropriate to determine actual proficiency.

As previously mentioned, other scales exist to assess debriefing in healthcare simulation. The “SHORT” scale was specifically developed to assess short debriefings, therefore filling an existing gap in debriefing assessment in healthcare education. We believe that the ease to use of our scale along with its detailed anchors makes it applicable to many simulation activities with short debriefings, either in a simulation center or in clinical facilities (in situ simulation), and even after real emergent situations if a consequent debriefing is done. Our scale is complementary to other previously published scales such as DASH [6] and OSAD [7], and we provide in Table 4 a comparison between their validity data.

**Table 4:** Comparison of scale validations between the SHORT, the DASH and the OSAD

Rating scales	SHORT	DASH	OSAD
Acronym signification	*Simulation in Healthcare retrOaction Rating Tool*	*Debriefing Assessment for Simulation in Healthcare*	*Objective Structured Assessment of Debriefing*
Specifically-aimed aimed debriefing length	Short (10–15 min)	Undefined length	Long (30–45 min)
Countries of origin	Canada, France	USA, Canada	UK, USA, Australia
Targeted educational population	General healthcare practitioners	General healthcare practitioners	Surgeons
Summary of the methodology of the scale design and validation	1. Existing literature review 2. Focus group to identify needs 3. Scale elaboration with experts 4. Pre-test on 2 debriefings 5. Rating of recorded debriefings 6. Assessment of reliability and validity	1. Existing Literature review 2. Iterative process of theory elaboration 3. Scale elaboration with experts 4. 5 h-webinars with international healthcare educators 5. Assessment of reliability and validity	1. Existing literature review 2. Semi-structured interviews 3. Scale elaboration with experts 4 Rating of recorded debriefings 5. Retest with a new rating of randomized recorded sessions 6 months later 6. Assessment of reliability and validity
Approach to validity and reliability measurement	Evidence of validity (test content, internal structure, testing consequences and response process) and reliability with generalizability theory: sources of variance for the scores, G and D studies with generalizability coefficients φ and φ-λ3	Reliability by interrater reliability (intraclass correlation coefficients) and internal consistency with Cronbach α. Validity by 1-way repeated-measures of variance	Reliability by inter-rater intraclass correlation coefficient (ICC) and test–retest ICC. Validity by face and content validation
Number of raters	2	114	2
Only expert raters assessing the scale?	Yes	No	Yes
Other raters assessing the scale than the authors?	No	Yes	No
Number of rated debriefings for each rater	22	3	20
Mean debriefings’ length	10:35	Unknown	Unknown
Type of scale	Weighted, criterion referenced behaviorally anchored rating scale	6-element, unweighted, criterion referenced behaviorally anchored rating scale (BARS)	5-point Likert rating scale
Number of items to be rated in the scale	6	6	8
Anchors to help rating each item?	Yes	Yes	Yes
Detailed notice for scale use	Yes	Yes	Yes
Estimated mean time to complete the scale	2 min	Unknown	5 min
Available translations of the scale	English, French	English, French, Japanese, German, Spanish	English
Broad scale testing before publishing	No	Yes	No

Evidence for **internal structure** of the SHORT scale is provided by the G study. High reliability is demonstrated by an absolute G coefficient at 0.80 [24, 25, 27]. Analyzing the items’ contribution to total variance provides further evidence. The items seem to measure different constructs (I = 12.0%). Furthermore, the interaction between items and debriefers (I × B = 13.0%) shows that the debriefers’ scores on a specific item differ, suggesting they have variable expertise in the competencies associated with each item of the scale. These results provide support to the validity of the SHORT scale.

Analysis of the percentages of variance attributed to each facet and to the interaction between facets (Table 2) provides conclusive evidence regarding the metric qualities of the scale. The largest source of variance is debriefers (B = 32.1%). This is expected and desirable, since debriefers are the objects of the measurement, i.e., those being assessed. As such, they do, and should, contribute most to the total variance of the score.

The variance component related to the observations, nested within debriefers (O:B = 13.5%), does not allow us to separate the specific effects of the observation from that of the interaction between observation and debriefer. It suggests either that the specific moment at which an assessment is made affects the result of the assessment or that the same debriefer can be assessed differently by the same rater on different occasions. This could be related to the difficulty of the specific debriefing; for example, a debriefing in which several concepts must be touched upon in a short time could reduce the debriefer’s performance compared to a debriefing centered on a single concept. The contribution of this component to overall variance, however, is small, and unlikely to introduce important biases.

There is an interaction effect between raters and observations (R × O:B = 5.3%): raters seem to assess the debriefings differently on different occasions, but the origin of this interaction is not clear. With observations being specific to each debriefer, it is possible that part of the variance linked to O:B is in fact linked to the interaction between those two facets. Indeed, it is impossible to draw a clear conclusion with the design used.

The other effects and interactions are insignificant or null (max. 0.2%). Specifically, the minimal variance related to raters (R = 0%) and to the interactions between raters and debriefers (R × B) and between raters and items (R × I) suggest that the raters do not add significant error to the measure.

The **testing consequences** evidence for validity were conclusive; the decision to offer additional training based on a cut-point of 3 on the scale is reliable (φ-λ_3_ ≥ 0.80). Furthermore, the D1 (1 rater, 2 observations) study demonstrated coefficients with acceptable generalizability (φ ≥ 0.75), whereas the D2 study (1 rater, 3 observations) demonstrated a high reliability (φ ≥ 0.80) compatible with high-stake exams [24, 25]. Thus, assessment of the debriefing as well as the decision to offer additional training are reliable when made by only one rater assessing at least two debriefings by a given debriefer.

The **response process** evidence for validity aims to determine that no errors were associated with the raters’ uses of the instrument. It is supported by a quality control process and familiarity of the raters with the assessment tool [23]. Indeed, the raters in this study were experts and designed the assessment tool. As such, they were very familiar with the SHORT, but this might not be the case in any given institution. Thus, the conclusions of our study, including the G and D study results, are limited to contexts where the raters have a similar level of expertise to those of this paper. Given that the SHORT was originally developed in French, we are aware that linguistic and cultural idiosyncrasies might limit its use in other cultural settings. To that end, like other behavioral rating instruments, specific training in the use of the SHORT should be provided to all raters before its final implantation. To assist this process, a detailed rater guide is provided in *the* Additional file 1. Training in the correct use of the “SHORT” scale could and should be offered to raters before its use. The DASH team has described one such training with the use of webinars [6], but e-learning with the use of videos or followed by live-debriefing ratings could also be used to train the raters and assess their familiarity with the scale.

In conclusion, despite some limitations, we provide evidence that the SHORT has a high reliability and good evidence for validity, although additional validity studies should be conducted, especially with raters that have not contributed to developing the instrument.

## Conclusion

We describe in this article the “Simulation in Healthcare retrOaction Rating Tool” (SHORT), a new instrument for assessing short debriefings that follow immersive simulation. It is our hope that the SHORT will be useful to healthcare educators wishing to assess their short debriefings or to determine the need for further debriefing training. Using GT, we were able to demonstrate a validity and reliability sufficient for its purpose, but additional studies should be done, especially to demonstrate the validity of the English version of the instrument and to validate its use by novice raters trained in the use of the SHORT.

## Additional file

Additional file 1: Appendix: SHORT scale and rater’s guide. (PDF 368 kb)

Additional file 2: GRILLE SHORT. (PDF 325 kb)

## Abbreviations

ANOVA: ANalysis Of VAriance
DASH: Debriefing Assessment for Simulation in Healthcare
GT: Generalizability Theory
OSAD: Objective Structures Assessment of Debriefing
PGY: Post-Graduate Year
SHORT: Simulation in Healthcare retrOaction Rating Tool
