# Supplementary material for: A new tool for assessing short debriefings after immersive simulation: validity of the SHORT scale
Source: BMC Med Educ. 2019 Mar 12;19:82. doi: 10.1186/s12909-019-1503-4 (PMC6419351; doi:10.1186/s12909-019-1503-4)
Supplement: Supplementary file 1 — Appendix: SHORT scale and rater’s guide. (PDF 368 kb) [file 12909_2019_1503_MOESM1_ESM.pdf]

UNIVERSITÉ LAVAL
Centre Apprentiss

GLOBAL RATING SCALE FOR ASSESSING SHORT DEBRIEFINGS

THE SHORT SCALE

SIMULATION IN HEALTHCARE RETROACTION RATING TOOL

université de BORDEAUX
Collège Sciences de la santé

Date and time of debriefing:	*Date of assessment:*
Case ID / debriefer's initials:	*Rater's initials:*

**SCORE** *(see holistic rating below)*	**1/5**	**2/5**	**3/5**	**4/5**	**5/5**
**DESCRIPTION**	Harmful	Neutral	Must improve	Could improve	Expert

Item 1: DEBRIEFING ENVIRONMENT (fosters a safe and effective debriefing environment with a positive tone)

1	2	3	4	5
Shows **no respect** for the learners and their emotional security	Shows **little respect** for the learners **OR** little concern for their **emotional safety**	Shows **respect** for the learners **AND** concern for their **emotional safety**	Shows **respect** for the learners **AND** concern for their **emotional safety**	Shows **respect** for the learners **AND** concern for their **emotional safety**
Does **NOT** help in reducing the learners' tension and stress	Helps **slightly** in reducing the learners' tension and stress	Helps **slightly** in reducing the learners' tension and stress	Helps **moderately** in reducing the learners' tension and stress	Helps **effectively** in reducing the learners' tension and stress

COMMENTS:

Item 2: DEBRIEFING STRUCTURE (organized structure)

1	2	3	4	5
Does **NOT** allow for an emotional phase	Facilitates a **short AND incomplete** emotional phase	Facilitates a **short AND incomplete** emotional phase	Facilitates a **fair** emotional phase	Facilitates an **excellent** emotional phase
Does **NOT** facilitate a summary at the end of the session	Does **NOT** facilitate a summary at the end of the session	Does **NOT** facilitate a summary at the end of the session	Facilitates an **adequate** summary at the end of the session	Facilitates an **effective** summary at the end of the session
Does **NOT** ensure that the debriefing meets the case objectives	Does **NOT** ensure that the debriefing meets the case objectives	**Ensures** that the debriefing meets the case objectives	**Ensures** that the debriefing meets the case objectives	**Ensures** that the debriefing meets the case objectives

COMMENTS:

Item 3: DEBRIEFING FACILITATION (efficiency and fluidity)

1	2	3	4	5
**Prevents** group discussion through **continuous** interventions	**Does NOT encourage** group discussion by intervening **excessively**	**Slightly encourages** group discussion despite **some excessive** interventions	**Regularly encourages** group discussion **without excessive** interventions	**Effectively encourages** group discussion **without excessive** interventions
Does **NOT reorient** discussions that digress from the objectives	**Reorients only slightly** the discussions that digress from the objectives	**Consistently tries to reorient** the discussions that digress from the objectives	**Reorient correctly** the discussions that digress from the objectives	**Reorients effectively** the discussions that digress from the objectives
Does **NOT manage** at all the resistant or disruptive learner (when applicable)	**Avoids managing** the resistant or disruptive learner (when applicable)	**Tries to manage** the resistant or disruptive learner (when applicable)	**Correctly manages** the resistant or disruptive learner (when applicable)	**Effectively manages** the resistant or disruptive learner (when applicable)

COMMENTS:

Item 4: ANALYSIS (facilitates an analysis of learner performance: « *Identify the gap* » - contextualizing)

1	2	3	4	5
Does **NOT discuss** the learners' performance in the context of their lived experience	Does **NOT encourage discussion** about the learners' **performance** in the context of their lived experience	**Encourages discussion** about the learners' **performance** in the context of their lived experience	**Encourages discussion** about the learners' **performance** in the context of their lived experience	**Encourages discussion** about the learners' **performance** in the context of their lived experience
Does **NOT explore** the cognitive frames underlying the learners' performance	Does **NOT explore** the cognitive frames underlying the learners' performance	Does **NOT explore** the cognitive frames underlying the learners' performance	**Explores** the cognitive frames underlying the learners' performance	**Explores effectively** the cognitive frames underlying the learners' performance

COMMENTS:

Item 5: TRANSFER (encourages learning transfer: « *close the gap* » - decontextualizing and recontextualizing)

1	2	3	4	5
Brings out **inaccurate** overarching principles	Does **NOT** bring out the **overarching principles** related to the case (knowledge and/or management strategies)	**Tries** to bring out the **overarching principles** related to the case (knowledge and/or management strategies)	**Brings out** the **overarching principles** related to the case (knowledge and/or management strategies)	**Brings out** the **overarching principles** related to the case (knowledge and/or management strategies)
Debriefs **only** based on abstract concepts	Debriefs **mostly** based on abstract concepts	Debriefs **mostly** based on abstract concepts	Causes the **emergence of concrete strategies** for management (rather than debriefing based on abstract principles)	Causes the **emergence of concrete strategies** for management (rather than debriefing based on abstract principles)
Does **NOT** project the learner in the context of a new situation (prototypal, similar)	Does **NOT** project the learner in the context of a new situation (prototypal, similar)	Does **NOT** project the learner in the context of a new situation (prototypal, similar)	Does **NOT** project the learner in the context of a new situation (prototypal, similar)	**Projects** the learner in the context of a new situation (prototypal, similar)

COMMENTS:

EXPERT GLOBAL SCORE: holistic assessment; NOT the average score

SCORE	1/5	2/5	3/5	4/5	5/5
**DESCRIPTION**	Harmful<br>*Undoes learning OR harms the credibility of the training or of the simulation modality*	Neutral<br>*No benefit from the debriefing OR does not make the simulation modality relevant*	Must improve<br>*Encourages learning, BUT does not allow the simulation modality to be used at its optimal capacity*	Could improve<br>*Encourages learning significantly AND allows the simulation modality to be used at its optimal capacity*	Expert<br>*Encourages learning significantly. Could be cited as an example OR the debriefer could train other debriefers*

NUMBER OF OBJECTIVES REACHED DURING THE CASE : ____ /____

Overall comments:

Authors : *Etienne Rivière MD, PhD, MA(Ed), Samuel-Lessard Tremblay, MD, Gilles Lortie, MD, PhD, & Gilles Chiniara, MD, MA(Ed).*
Adapted from *« Évaluation du débreffage au centre Apprentiss » by Gilles Chiniara, and debriefing evaluation by M Jaffrelot & G Savoldelli, 2010, inspired from the DASH© evaluation scale (Brett-Fleegler M. et al, Simulation in Healthcare, 2009).*

RATER'S GUIDE TO USE THE SHORT SCALE

The SHORT instrument includes a global rating scale composed of 5 items and a holistic expert evaluation.

Each of the 5 items is scored on a scale from 1 (harmful) to 5 (expert), using specific **cues** provided to guide scoring. The cues for each item are placed on separate lines based on their topic or theme. For each item, rate each line individually, then rate the item. If the lines of a single item give different scores, the highest score should be adopted for the item. However, for some items, specific lines should weigh more in the final item score; such weighing is described in the rating guide.

The holistic evaluation is rated on a scale from 1 to 5 as follows. Please note that the **holistic evaluation should not be based on the mean score of the five preceding items.**

- "1 = harmful", *i.e.* undoes learning, or harms the credibility of the training or of the simulation modality;
- "2 = neutral", *i.e.* learners gain no benefit from the debriefing, or the debriefer does not make the simulation modality relevant;
- "3 = must improve", *i.e.* the debriefer encourages learning, but does not allow the simulation modality to be used at its optimal capacity;
- "4 = could improve", *i.e.* the debriefer encourages learning significantly and allows the simulation modality to be used at its optimal capacity;
- "5 = expert", *i.e.* the debriefer encourages learning significantly and could be cited as an example, or the debriefer could train other debriefers.

EXAMPLE:

The cue lines help the rater to score the debriefer as accurately as possible. Each cue line should be rated independently, then the item should be scored based on the full lines that delineate successive cues:

Item 1 : ENVIRONMENT (create a convivial environment with a positive tone encouraging effective debriefing)

1	2	3	4	5
Demonstrate **no respect** of the learners and for their emotional security	Demonstrate **little respect** of the learners **OR** little concern for their **emotional security**	Demonstrate **respect** of the learners **AND** concern for their **emotional security**	Demonstrate **respect** of the learners **AND** concern for their **emotional security**	Demonstrate **respect** of the learners **AND** concern for their **emotional security**
Do **NOT** contribute to reduce tension and stress of the learners	Contribute **slightly** to reduce tension and stress of the learners	Contribute **slightly** to reduce tension and stress of the learners	Contribute **moderately** to reduce tension and stress of the learners	Contribute **effectively** to reduce tension and stress of the learners

Global rating for the item = **3**/5

In case of large discrepancies between different cue line scores for an item, a mean score should be averaged, except in items where the cue lines are weighed differently, as specified in the individual item descriptions below.

A score of 3 or less on any item or on the holistic evaluation should prompt consideration of providing additional training on relevant aspects of the debriefer's performance.

<u>How to rate each of the 5 items</u>

D = DEBRIEFER

• ITEM 1: ENVIRONMENT

GOAL: To foster a safe and effective debriefing environment with a positive tone that encourages effective debriefing

1.1. Respect and emotional security

<table>
<tr><th>RATING</th><th>1</th><th>2</th><th>3</th><th>4</th><th>5</th></tr>
<tr><td>CUE(S)</td><td>Shows no respect for the learners and their emotional security</td><td>Shows little respect for the learners OR little concern for their emotional safety</td><td>Shows respect for the learners AND concern for their emotional safety</td><td>Shows respect for the learners AND concern for their emotional safety</td><td>Shows respect for the learners AND concern for their emotional safety</td></tr>
<tr><td>EXAMPLES</td><td>- Undue arguing with learners
- Overt learner bashing
- Shaming</td><td>- No active efforts to ensure emotional security
- Neutral attitude
- Speech interruption</td><td colspan="3">- Active efforts to ensure emotional security
- Watchful supervision
- No or few speech interruption(s)
- Supportive attitude</td></tr>
</table>

1.2. Tension and stress management

<table>
<tr><th>RATING</th><th>1</th><th>2</th><th>3</th><th>4</th><th>5</th></tr>
<tr><td>CUE(S)</td><td>Does NOT help in reducing the learners' tension and stress</td><td>Helps slightly in reducing the learners' tension and stress</td><td>Helps slightly in reducing the learners' tension and stress</td><td>Helps moderately in reducing the learners' tension and stress</td><td>Helps effectively in reducing the learners' tension and stress</td></tr>
<tr><td>EXAMPLES</td><td>- Anger
- Accusations
- Making the learners feel guilty</td><td colspan="2">- Passive / neutral attitude
- Absence of normalisation of tension and stress</td><td>- Passive efforts to reduce tension (only by learners, but respected by D)
- Aborted or incomplete normalisation of tension and stress</td><td>- Active efforts to reduce tension
- Appropriate and complete normalisation of tension and stress</td></tr>
</table>

• ITEM 2: STRUCTURE

GOAL: To structure the debriefing to allow for discussion of central concepts relative to the case

2.1. Quality of the emotional phase

RATING	1	2	3	4	5
**CUE(S)**	Does **NOT** allow for an emotional phase	Facilitates a **short AND incomplete** emotional phase	Facilitates a **short AND incomplete** emotional phase	Facilitates a **fair** emotional phase	Facilitates an **excellent** emotional phase
**EXAMPLES**	Omission of the phase which allows learners to verbalize their emotions	Emotional phase interrupted by D<br>Or interruption by learners not controlled		Passive control over the emotional phase (by learners, but respected by D without interruption)	Active control over the emotional phase, with eventual redirection or focus of learners' emotions on a concept planned for discussion

2.2. Summary of concepts discussed during debriefing

RATING	1	2	3	4	5
**CUE(S)**	Does **NOT** facilitate a summary at the end of the session	Does **NOT** facilitate a summary at the end of the session	Does **NOT** facilitate a summary at the end of the session	Facilitates an **adequate** summary at the end of the session	Facilitates an **effective** summary at the end of the session
**EXAMPLES**	Omission of a summary of concepts discussed in the debriefing			Summary too brief, or interrupted, or incomplete	Efficient summary of concepts discussed during debriefing, with verbalization of transfer of these concepts to a future case, either simulated or not

2.3. Targeting learning outcomes defined for the case

RATING	1	2	3	4	5
**CUE(S)**	Does **NOT** ensure that the debriefing meets the case objectives	Does **NOT** ensure that the debriefing meets the case objectives	**Ensures** that the debriefing meets the case objectives	**Ensures** that the debriefing meets the case objectives	**Ensures** that the debriefing meets the case objectives
**EXAMPLES**	D has obviously not reviewed specific learning outcomes for the case in order to target them during debriefing		D has obviously reviewed specific learning outcomes for the case and targets all or most of them during debriefing		

2.4. Weighing

In case of a rating contradiction between the three lines, the presence of a summary should be a priority, then the presence of an efficient emotional phase and then targeted learning outcomes. The presence of an effective summary that allows learning transfer to occur is a hallmark of debriefing expertise.

• ITEM 3: ANIMATION

GOAL: To ensure efficiency and fluidity of the debriefing

3.1. Guiding group discussion

RATING	1	2	3	4	5
**CUE(S)**	**Prevents** group discussion through **continuous** interventions	**Does NOT encourage** group discussion by intervening **excessively**	**Slightly encourages** group discussion despite **some excessive** interventions	**Regularly encourages** group discussion **without excessive** interventions	**Effectively encourages** group discussion **without excessive** interventions
**EXAMPLES**	- D talks exclusively<br>- No or inadequate answers to learners' questions	- D talks most of the time with only little time for learners<br>- Few answers are given to learner's questions, some questions remaining unanswered	- Passive encouragement of discussion through a positive tone and environment with or without excessive interventions<br>- Active encouragement of discussion (with verbal or non-verbal actions), but with excessive interventions	- Active encouragement of discussion (with verbal or non-verbal actions), without excessive interventions	- Active encouragement of discussion (with verbal or non-verbal actions)<br>- D lead the discussion efficiently, targeting all learning outcomes

3.2. Redirection of digressive objectives

RATING	1	2	3	4	5
**CUE(S)**	Does **NOT reorient** discussions that digress from the objectives	**Reorients only slightly** the discussions that digress from the objectives	**Consistently tries to reorient** the discussions that digress from the objectives	**Reorient correctly** the discussions that digress from the objectives	**Reorients effectively** the discussions that digress from the objectives
**EXAMPLES**	- D talks exclusively, leaving no time for learners' discussion	- D initiates a reorientation of digressing discussions, but fail to do so and gives up	- D initiates a reorientation of digressing discussions and fails to do so, but continues to try	- D partly succeeds in reorienting digressing discussions	- D fully succeeds in reorienting digressing discussions

3.3. Managing the resistant or disruptive learner

RATING	1	2	3	4	5
**CUE(S)**	Does **NOT manage** at all the resistant or disruptive learner (when applicable)	**Avoids managing** the resistant or disruptive learner (when applicable)	**Tries to manage** the resistant or disruptive learner (when applicable)	**Correctly manages** the resistant or disruptive learner (when applicable)	**Effectively manages** the resistant or disruptive learner (when applicable)
**EXAMPLES**	- D refuses to manage the resistant or disruptive learner<br>- D does not recognize a resistant or disruptive learner	- D recognizes a resistant or disruptive learner, but avoids tackling this issue	- D initiates efforts to manage a resistant or disruptive learner, but fails to do so	- D partially manages a resistant or disruptive learner and dedicates minimal time to that end	- D makes adequate efforts to manage a resistant or disruptive learner and allows adequate time to that end

3.4. Weighing

Most of the time, if the first line is scored 1 or 2, the other lines are not applicable and should not be rated. Thus, the final score for the item is that of the first line.

• Item 4: ANALYSIS

Goal: To animate the analysis of learners' performance and « *identify the knowledge gap* » - contextualizing

4.1. Ability to discuss the learners' performance

RATING	1	2	3	4	5
**CUE(S)**	Does **NOT discuss** the learners' performance in the context of their lived experience	Does **NOT encourage discussion** about the learners' **performance** in the context of their lived experience	**Encourages discussion** about the learners' **performance** in the context of their lived experience	**Encourages discussion** about the learners' **performance** in the context of their lived experience	**Encourages discussion** about the learners' **performance** in the context of their lived experience
**EXAMPLES**	- D completely and/or deliberately omits to rely on learners' performance during the simulated case<br>- D dedicates all the debriefing time to a lecture	- D relies partially on learner's performance during the simulated case	- D fully relies on learner's performance during the simulated case<br>- D encourages learners to explain what happened during the simulated case through their own eyes<br>- D encourages all participants to verbalize their feelings and cognitive frames		

4.2. Exploration of learner's cognitive frames

RATING	1	2	3	4	5
**CUE(S)**	Does **NOT explore** the cognitive frames underlying the learners' performance	Does **NOT explore** the cognitive frames underlying the learners' performance	Does **NOT explore** the cognitive frames underlying the learners' performance	**Explores** the cognitive frames underlying the learners' performance	**Explores effectively** the cognitive frames underlying the learners' performance
**EXAMPLES**	- D omits or avoids exploring cognitive frames<br>- Passive exposure of cognitive frames by learners<br>- Absence of confrontation of learners' cognitive frames with D's own			- Incomplete or inadequate (judgmental or Socratic) exploration of cognitive frames<br>- Confrontation of learner's cognitive frames with D's own	- Complete and non-judgmental exploration of cognitive frames<br>- Confrontation of learner's cognitive frames with D's own

- **Item 5: transfer**

Goal: To encourage learning transfer and « *close the knowledge gap* » - decontextualizing and recontextualizing

5.1. Ability to bring out overarching principles related to the learners' knowledge gap (decontextualizing)

<table>
<tr><th>RATING</th><th>1</th><th>2</th><th>3</th><th>4</th><th>5</th></tr>
<tr><td>CUE(S)</td><td>Brings out inaccurate overarching principles</td><td>Does NOT bring out the overarching principles related to the case (knowledge and/or management strategies)</td><td>Tries to bring out the overarching principles related to the case (knowledge and/or management strategies)</td><td>Brings out the overarching principles related to the case (knowledge and/or management strategies)</td><td>Brings out the overarching principles related to the case (knowledge and/or management strategies)</td></tr>
<tr><td>EXAMPLES</td><td>- Wrong general principles
- Outdated principles</td><td>- No mention of overarching principles adapted to learners' performance</td><td>- Overarching principles partially adapted to learners' performance
- D allows learners to partially close their knowledge gap</td><td colspan="2">- Overarching principles fully adapted to learners' performance
- D allows learners to fully close their knowledge gap
- Learners are now equipped with general principles that could be mobilized in new situations, either similar or prototypal</td></tr>
</table>

5.2. Ability to bring out concrete strategies relative to learner's knowledge gap (decontextualizing)

<table>
<tr><th>RATING</th><th>1</th><th>2</th><th>3</th><th>4</th><th>5</th></tr>
<tr><td>CUE(S)</td><td>Debriefs only based on abstract concepts</td><td>Debriefs mostly based on abstract concepts</td><td>Debriefs mostly based on abstract concepts</td><td>Causes the emergence of concrete strategies for management (rather than debriefing based on abstract principles)</td><td>Causes the emergence of concrete strategies for management (rather than debriefing based on abstract principles)</td></tr>
<tr><td>EXAMPLES</td><td>- D dedicates all the debriefing time to a lecture, without any concrete strategies</td><td colspan="2">- D dedicates some time to a lecture
- Few or very few concrete strategies and/or not adapted to the lived experience</td><td colspan="2">- D facilitates the discussion of concrete strategies appropriate to management of a similar case in clinical practice and adapted to the lived experience</td></tr>
</table>

5.3. Ability to project learners in a new situation either prototypal or similar (recontextualizing)

<table>
<tr><th>RATING</th><th>1</th><th>2</th><th>3</th><th>4</th><th>5</th></tr>
<tr><td>CUE(S)</td><td>Does NOT project the learner in the context of a new situation (prototypal, similar)</td><td>Does NOT project the learner in the context of a new situation (prototypal, similar)</td><td>Does NOT project the learner in the context of a new situation (prototypal, similar)</td><td>Does NOT project the learner in the context of a new situation (prototypal, similar)</td><td>Projects the learner in the context of a new situation (prototypal, similar)</td></tr>
<tr><td>EXAMPLES</td><td colspan="4">- D does not project learners into a new situation during debriefing
- D does not make the prototypical aspects of the case explicit or does not ask learners about the prototypal aspect of the case</td><td>- D makes explicit the prototypal aspect of the case or asks learners about them, then guides learners in applying their knowledge to a new similar situation (or a prototypal situation if this was not the case) discussed during debriefing</td></tr>
</table>

5.4. Weighing

If scoring highlights discordance between the lines, then recontextualizing (line 3) should weigh more heavily for the item score, followed by concrete strategies (line 2), and finally overarching principles (line 1).
