# Supplementary material for: A new tool for assessing short debriefings after immersive simulation: validity of the SHORT scale
Source: BMC Med Educ. 2019 Mar 12;19:82. doi: 10.1186/s12909-019-1503-4 (PMC6419351; doi:10.1186/s12909-019-1503-4)
Supplement: Supplementary file 2 — GRILLE SHORT. (PDF 325 kb) [file 12909_2019_1503_MOESM2_ESM.pdf]

UNIVERSITÉ LAVAL
Centre Apprentiss

GRILLE D'EVALUATION DES DEBREFFAGES COURTS

# GRILLE SHORT

*SIMULATION IN HEALTHCARE RETROACTION RATING TOOL*

université de BORDEAUX
Collège Sciences de la santé

*Date et heure de la séance :* *Date d'évaluation :*

*Identification du cas / initiales du débreffeur :* *Initiales de l'évaluateur :*

| SCORE (voir l'évaluation holistique plus bas) | 1/5 | 2/5 | 3/5 | 4/5 | 5/5 |
|---|---|---|---|---|---|
| DESCRIPTION | Délétère | Neutre | Doit s'améliorer | Peut s'améliorer | Expert |

### Item 1 : ENVIRONNEMENT (crée un environnement de débreffage efficace et convivial : « *ton* »)

| 1 | 2 | 3 | 4 | 5 |
|---|---|---|---|---|
| Ne démontre **aucun respect** pour les apprenants et leur **sécurité affective** | Démontre **peu de respect** pour les apprenants **ou** peu de souci pour leur **sécurité affective** | Démontre un **respect** pour les apprenants **et** un souci de leur **sécurité affective** | Démontre un **respect** pour les apprenants **et** un souci de leur **sécurité affective** | Démontre un **respect** pour les apprenants **et** un souci de leur **sécurité affective** |
| **Ne contribue pas** à la baisse de la tension et du stress | Contribue **peu** à la baisse de la tension et du stress | Contribue **peu** à la baisse de la tension et du stress | Diminue **assez** la tension et le stress | Diminue **efficacement** la tension et le stress |

**COMMENTAIRES :**

### Item 2 : ORGANISATION du DÉBREFFAGE (de façon organisée : « *structure* »)

| 1 | 2 | 3 | 4 | 5 |
|---|---|---|---|---|
| **Ne fait pas ventiler** les émotions des apprenants | **Fait peu ventiler** les émotions des apprenants | **Fait peu ventiler** les émotions des apprenants | Fait une **assez bonne** ventilation des émotions des apprenants | Fait une **excellente** ventilation des émotions des apprenants |
| N'anime **pas** de **sommaire des apprentissages** en fin de session | N'anime **pas** de **sommaire des apprentissages** en fin de session | N'anime **pas** de **sommaire des apprentissages** en fin de session | Anime un **sommaire adéquat des apprentissages** en fin de session | Anime un **sommaire efficace des apprentissages** en fin de session |
| Ne s'assure **pas** que le débreffage **remplit les objectifs** du cas | Ne s'assure **pas** que le débreffage **remplit les objectifs** du cas | S'assure que le débreffage **remplit les objectifs** du cas | S'assure que le débreffage **remplit les objectifs** du cas | S'assure que le débreffage **remplit les objectifs** du cas |

**COMMENTAIRES :**

### Item 3 : ANIMATION du DÉBREFFAGE (de façon efficace : « *fluidité* »)

| 1 | 2 | 3 | 4 | 5 |
|---|---|---|---|---|
| **Empêche** la discussion de groupe par ses interventions **continues** | N'encourage **pas** la discussion de groupe **en faisant plusieurs interventions excessives** | Encourage **légèrement** la discussion de groupe **mais fait quelques interventions excessives** | Encourage **souvent** la discussion de groupe **sans interventions excessives** | Encourage **efficacement** la discussion de groupe **sans interventions excessives** |
| **Ne réoriente pas** les discussions qui s'écartent des objectifs | **Ne réoriente que peu** les discussions qui s'écartent des objectifs | **Essaie de réorienter** les discussions qui s'écartent des objectifs | **Réoriente correctement** les discussions qui s'écartent des objectifs | Réoriente **efficacement** les discussions qui s'écartent des objectifs |
| **Ne gère pas** l'apprenant **réfractaire** *(si applicable)* | **Evite** de gérer l'apprenant **réfractaire** *(si applicable)* | **Essaie** de gérer l'apprenant **réfractaire** *(si applicable)* | Gère **correctement** l'apprenant **réfractaire** *(si applicable)* | Gère **efficacement** l'apprenant **réfractaire** *(si applicable)* |

**COMMENTAIRES :**

### Item 4 : ANALYSE (Anime l'analyse de la performance des apprenants : « *identifie les lacunes* »)

| 1 | 2 | 3 | 4 | 5 |
|---|---|---|---|---|
| **Ne discute pas** de la **performance** des apprenants dans le contexte du cas vécu | N'encourage **pas** de discussion de la **performance** des apprenants dans le contexte du cas vécu | Encourage une discussion de la **performance** des apprenants dans le contexte du cas vécu | Encourage une discussion de la **performance** des apprenants dans le contexte du cas vécu | Encourage une discussion de la **performance** des apprenants dans le contexte du cas vécu |
| N'explore **pas** les **processus cognitifs sous-jacents** à la performance des apprenants | N'explore **pas** les **processus cognitifs sous-jacents** à la performance des apprenants | N'explore **pas** les **processus cognitifs sous-jacents** à la performance des apprenants | Explore les **processus cognitifs sous-jacents** à la performance des apprenants | Explore **efficacement** les **processus cognitifs sous-jacents** à la performance des apprenants |

**COMMENTAIRES :**

© **Grille SHORT** : évaluation des débreffages courts – version originale du 29 juillet 2016 – centre Apprentiss, Université Laval, Québec, Canada.

**Item 5 : TRANSFERT (encourage le transfert des connaissances : « *comble les lacunes* »)**

| 1 | 2 | 3 | 4 | 5 |
|---|---|---|---|---|
| Fait émerger des **principes généraux erronés** compte tenu de la situation vécue | **Ne fait pas** émerger les **principes généraux** relatifs à la situation vécue (connaissances et/ou stratégies de prise en charge) | **Essaie de faire** émerger les **principes généraux** relatifs à la situation vécue (connaissances et/ou stratégies de prise en charge) | Fait émerger les **principes généraux** relatifs à la situation vécue (connaissances et/ou stratégies de prise en charge) | Fait émerger les **principes généraux** relatifs à la situation vécue (connaissances et/ou stratégies de prise en charge) |
| Ne débreffe **que** sur des points purement **abstraits** | Débreffe plutôt sur des points purement **abstraits** | Débreffe plutôt sur des points purement **abstraits** | Provoque **l'émergence de stratégies concrètes de prise en charge** (pas de débreffage sur des points abstraits) | Provoque **l'émergence de stratégies concrètes de prise en charge** (pas de débreffage sur des points abstraits) |
| **Ne replace pas** l'apprenant en situation nouvelle (prototypique, similaire) | **Ne replace pas l**'apprenant en situation nouvelle (prototypique, similaire) | **Ne replace pas** l'apprenant en situation nouvelle (prototypique, similaire) | **Ne replace pas** l'apprenant en situation nouvelle (prototypique, similaire) | **Replace** l'apprenant en situation nouvelle (prototypique, similaire) |

**COMMENTAIRES :**

## EVALUATION GLOBALE de l'EXPERT : Évaluation holistique, ne pas faire de moyenne !

| **SCORE** | **1/5** | **2/5** | **3/5** | **4/5** | **5/5** |
|---|---|---|---|---|---|
| **DESCRIPTION** | Nocif<br>*Désapprend ou nuit à la crédibilité de la formation ou de la modalité de simulation* | Neutre<br>*Pas de bénéfice apporté par le débreffage ou ne rend pas l'utilisation de la modalité de simulation pertinente* | Doit s'améliorer<br>*Apprentissage favorisé, mais ne permet pas une utilisation optimale de la modalité de simulation* | Peut s'améliorer<br>*Apprentissage favorisé de manière significative et rend optimale l'utilisation la modalité de simulation* | Expert<br>*Apprentissage significatif. Pourrait être cité en exemple, ou le débreffeur pourrait être amené à former d'autres débreffeurs* |

**NOMBRE D'OBJECTIFS ATTEINTS POUR LE CAS : ____ /____**

Commentaires généraux :

***Auteurs :*** *Etienne Rivière MD, PhD, MA(Ed), Samuel-Lessard Tremblay, MD, Gilles Lortie, MD, PhD, & Gilles Chiniara, MD, MA(Ed).*
*D'après la grille conçue pour « l'évaluation du débreffage au centre Apprentiss » par Gilles Chiniara, et l'évaluation des débreffages de M Jaffrelot & G Savoldelli, 2010, inspirée de l'échelle DASH© (Brett-Fleegler M. et al, Simulation in Healthcare, 2009).*

# GUIDE D'UTILISATION DE LA GRILLE SHORT

La grille SHORT est une grille utilisant une échelle d'évaluation globale (ou *global rating scale*) constituée de 5 items ainsi que d'une évaluation holistique experte.

Chacun des 5 items est noté de 1 (délétère à l'apprentissage) à 5 (expert), en utilisant les indices spécifiques visant à guider la notation. Pour chaque item, il faut noter chaque ligne individuellement, puis noter l'item. Si les notes obtenues à chaque ligne d'un même item sont différentes, retenez la note la plus élevée pour l'item. Cependant, pour certains items, certaines lignes ont une pondération plus élevée dans la note finale de l'item. Ces pondérations spécifiques sont détaillées au fur et à mesure de la description des items ci-après.

L'évaluation holistique d'expert est une note de 1 à 5 comme suit. Notez bien qu'il ne s'agit pas là d'une moyenne des notes obtenues à chacun des 5 items précédents.

- « 1 = délétère », c'est-à-dire que le débreffeur amène à un contre apprentissage ou un «désapprentissage», ou nuit à la crédibilité de la formation, ou à la crédibilité de l'utilisation de l'outil qu'est la simulation pour l'atteinte des cibles d'apprentissage fixées ;
- « 2 = neutre », c'est-à-dire que les apprenants ne tirent aucun bénéfice du débreffage, ou que le débreffeur ne tire aucun profit de l'utilisation de la simulation pour les apprentissages ciblés ;
- « 3 = doit s'améliorer », c'est-à-dire que le débreffeur permet un peu l'apprentissage mais ne tire pas tous les bénéfices de l'utilisation de la simulation pour atteindre les cibles d'apprentissage fixées ;
- « 4 = peut s'améliorer », c'est-à-dire que le débreffeur permet des apprentissages significatifs en employant adéquatement la simulation pour atteindre les cibles d'apprentissage fixées ;
- « 5 = expert », c'est-à-dire que le débreffeur permet de nombreux apprentissages en utilisant la simulation de manière optimale, et pourrait être cité à titre d'exemple, ou pourrait former d'autres débreffeurs.

Un score de 3 ou moins à tout item ou à l'évaluation holistique doit conduire à la mise en marche de formations générales ou ciblées pour développer les compétences du débreffeur en matière de débreffage, et le faire progresser au niveau 4 ou 5.

**Exemple de notation d'un item (ici l'item 1/5 : « environnement »)**
Les lignes d'indices guident l'évaluateur dans sa notation pour qu'elle soit la plus précise possible. Chaque ligne doit être notée de manière indépendante, puis la note globale pour l'item est définie en tenant compte des lignes délimitant chaque indice, par exemple :

**Item 1 : ENVIRONNEMENT (crée un environnement de débreffage efficace et convivial : « *ton* »)**

| 1 | 2 | 3 | 4 | 5 |
|---|---|---|---|---|
| Ne démontre **aucun respect** pour les apprenants et leur **sécurité affective** | Démontre **peu de respect** pour les apprenants **ou** peu de souci pour leur **sécurité affective** | Démontre un **respect** pour les apprenants **et** un souci de leur **sécurité affective** | Démontre un **respect** pour les apprenants **et** un souci de leur **sécurité affective** | Démontre un **respect** pour les apprenants **et** un souci de leur **sécurité affective** |
| **Ne contribue pas** à la baisse de la tension et du stress | Contribue **peu** à la baisse de la tension et du stress | Contribue **peu** à la baisse de la tension et du stress | Diminue **assez** la tension et le stress | Diminue **efficacement** la tension et le stress |

**Note globale de l'item : 3/5**

En cas de différences majeures entre les notes d'un même item, une note moyenne est attribuée, sauf pour les items comportant une ligne d'indices à plus forte pondération, comme précisé plus bas dans le descriptif détaillé de la notation des items.

La notation holistique doit être considérée comme indépendante : c'est une grille à part entière. Pour rappel, elle ne doit pas être la moyenne de la notation des 5 items précédents.

# <u>Comment noter chacun des 5 items</u>

**D = DEBREFFEUR**

## • ITEM 1: ENVIRONNEMENT

**OBJECTIF : Promouvoir un climat de sécurité affective permettant un apprentissage efficace et une meilleure participation des apprenants au débreffage**

### 1.1. Respect de la sécurité affective

| NOTATION | 1 | 2 | 3 | 4 | 5 |
|---|---|---|---|---|---|
| **INDICE(S)** | Ne démontre **aucun respect** pour les apprenants et leur **sécurité affective** | Démontre **peu de respect** pour les apprenants **ou** peu de souci pour leur **sécurité affective** | Démontre un **respect** pour les apprenants **et** un souci de leur **sécurité affective** | Démontre un **respect** pour les apprenants **et** un souci de leur **sécurité affective** | Démontre un **respect** pour les apprenants **et** un souci de leur **sécurité affective** |
| **EXEMPLES** | - Ton inapproprié<br>- Dispute<br>- Humilie ou rabroue les apprenants | - Aucun effort actif pour assurer la sécurité affective<br>- Attitude neutre<br>- Interruptions régulières des apprenants | - Efforts actifs pour assurer la sécurité affective des apprenants<br>- Supervision attentive<br>- Pas ou peu d'interruptions des apprenants<br>- Attitude de soutien | | |

### 1.2. Tension et gestion du stress

| NOTATION | 1 | 2 | 3 | 4 | 5 |
|---|---|---|---|---|---|
| **INDICE(S)** | **Ne contribue pas** à la baisse de la tension et du stress | Contribue **peu** à la baisse de la tension et du stress | Contribue **peu** à la baisse de la tension et du stress | Diminue **assez** la tension et le stress | Diminue **efficacement** la tension et le stress |
| **EXEMPLES** | - Colère<br>- Accusations<br>- Provoque une culpabilité chez les apprenants | - Attitude passive ou neutre<br>- Absence de normalisation de la tension et du stress | | - Efforts passifs pour réduire la tension (seulement par les apprenants, mais respecté par le D)<br>- Tentative avortée ou incomplète de réduction du stress des apprenants | - Efforts actifs pour réduire la tension<br>- Réduction complète et appropriée du stress des apprenants |

## • ITEM 2: ORGANISATION

**OBJECTIF: Structurer le débreffage pour permettre la discussion des concepts majeurs relatifs à la situation vécue**

### 2.1. Qualité de la phase émotive

| NOTATION | 1 | 2 | 3 | 4 | 5 |
|---|---|---|---|---|---|
| **INDICE(S)** | **Ne fait pas ventiler** les émotions des apprenants | **Fait peu ventiler** les émotions des apprenants | **Fait peu ventiler** les émotions des apprenants | Fait une **assez bonne** ventilation des émotions des apprenants | Fait une **excellente** ventilation des émotions des apprenants |
| **EXEMPLES** | Omission de la phase émotive | Phase émotive interrompue par le D, ou interruptions par les apprenants non contrôlées par le D | | Contrôle passif de la phase émotive (par les apprenants, respect de la phase par le D sans interruption) | Contrôle actif de la phase émotive avec redirection éventuelle des apprenants sur la verbalisation de leurs émotions, ou la focalisation sur un concept majeur à traiter ultérieurement pendant le débreffage |

### 2.2. Sommaire des concepts abordés pendant le débreffage

| NOTATION | 1 | 2 | 3 | 4 | 5 |
|---|---|---|---|---|---|
| **INDICE(S)** | N'anime **pas** de **sommaire des apprentissages** en fin de session | N'anime **pas** de **sommaire des apprentissages** en fin de session | N'anime **pas** de **sommaire des apprentissages** en fin de session | Anime un **sommaire adéquat des apprentissages** en fin de session | Anime un **sommaire efficace des apprentissages** en fin de session |
| **EXEMPLES** | Omission du sommaire | | | Sommaire trop bref, interrompu ou incomplet | Sommaire efficient des concepts abordés pendant le débreffage, avec initiation d'un transfert des apprentissages vers une situation similaire simulée ou non |

### 2.3. Atteinte des cibles d'apprentissage définies pour le cas

| NOTATION | 1 | 2 | 3 | 4 | 5 |
|---|---|---|---|---|---|
| **INDICE(S)** | Ne s'assure **pas** que le débreffage **remplit les objectifs** du cas | Ne s'assure **pas** que le débreffage **remplit les objectifs** du cas | S'assure que le débreffage **remplit les objectifs** du cas | S'assure que le débreffage **remplit les objectifs** du cas | S'assure que le débreffage **remplit les objectifs** du cas |
| **EXEMPLES** | Le D n'atteint pas les cibles d'apprentissage, ou ne les a manifestement pas consultées avant l'activité pour tenter de les atteindre | | Le D a consulté les cibles d'apprentissage avant l'activité et atteint toutes, ou presque toutes, ces cibles pendant le débreffage | | |

### 2.4. Pondération

En cas de notes contradictoires entre les 3 lignes, la présence d'un sommaire des apprentissages effectués doit être prioritaire, devant la présence d'une phase émotive. La ligne sur l'atteinte des cibles d'apprentissage est la moins contributive à la note. La présence d'un sommaire efficient prépare le transfert des apprentissages effectués pendant l'activité de simulation vers une nouvelle situation, et constitue un marqueur d'expertise du débreffeur.

## • ITEM 3: ANIMATION

**OBJECTIF: Assurer l'efficacité et la fluidité du débreffage**

### 3.1. Maîtrise de la discussion de groupe

| NOTATION | 1 | 2 | 3 | 4 | 5 |
|---|---|---|---|---|---|
| INDICE(S) | **Empêche** la discussion de groupe par ses interventions **continues** | N'encourage **pas** la discussion de groupe **en faisant plusieurs interventions excessives** | Encourage **légèrement** la discussion de groupe **mais fait quelques interventions excessives** | Encourage **souvent** la discussion de groupe **sans interventions excessives** | Encourage **efficacement** la discussion de groupe **sans interventions excessives** |
| EXEMPLES | - Le D ne fait que parler<br>- Peu ou pas de réponses aux questions des apprenants, ou des réponses inadéquates à leurs questions | - Le D parle la plupart du temps, n'en laissant que peu aux apprenants<br>- Seulement quelques réponses sont données aux questions des apprenants, avec quelques questions laissées sans réponse | - Encouragements passifs à la discussion grâce à un ton positif et un environnement avec ou sans interventions excessives<br>- Encouragements actifs à la discussion (langage verbal ou non verbal), mais interventions excessives | Encouragements actifs à la discussion (langage verbal ou non verbal), sans interventions excessives | - Encouragements actifs à la discussion (langage verbal ou non verbal)<br>- Le D conduit la discussion de manière efficace en atteignant toutes les cibles d'apprentissage |

### 3.2. Réorientation de la discussion

| NOTATION | 1 | 2 | 3 | 4 | 5 |
|---|---|---|---|---|---|
| INDICE(S) | **Ne réoriente pas** les discussions qui s'écartent des objectifs | **Ne réoriente que peu** les discussions qui s'écartent des objectifs | **Essaie de réorienter** les discussions qui s'écartent des objectifs | **Réoriente correctement** les discussions qui s'écartent des objectifs | Réoriente **efficacement** les discussions qui s'écartent des objectifs |
| EXEMPLES | Le D ne fait que parler, sans laisser de temps à la discussion avec les apprenants | Le D initie une réorientation de la discussion quand celle-ci s'écarte des objectifs, mais sans y parvenir et en abandonnant la tentative. | Le D initie une réorientation de la discussion quand celle-ci s'écarte des objectifs, mais n'y parvient pas, sans pour autant abandonner la tentative | Le D réussit partiellement à réorienter la discussion quand celle-ci s'écarte des objectifs, en persévérant | Le D réussit parfaitement à réorienter la discussion quand celle-ci s'écarte des objectifs |

### 3.3. Gestion d'un apprenant réfractaire

| NOTATION | 1 | 2 | 3 | 4 | 5 |
|---|---|---|---|---|---|
| INDICE(S) | **Ne gère pas** l'apprenant **réfractaire** *(si applicable)* | **Evite** de gérer l'apprenant **réfractaire** *(si applicable)* | **Essaie** de gérer l'apprenant **réfractaire** *(si applicable)* | Gère **correctement** l'apprenant **réfractaire** *(si applicable)* | Gère **efficacement** l'apprenant **réfractaire** *(si applicable)* |
| EXEMPLES | - Le D refuse de prendre en compte un apprenant réfractaire<br>- Ou le D ne reconnaît pas l'apprenant réfractaire | Le D reconnaît un apprenant réfractaire mais n'en tient pas compte dans le débreffage | Le D initie une démarche de prise en charge de l'apprenant réfractaire sans pour autant y parvenir | Le D parvient à prendre en charge un apprenant réfractaire, mais de manière incomplète et avec un temps dédié minimal | Le D fait les efforts nécessaire pour prendre adéquatement en charge un apprenant réfractaire, en optimisant son temps pour cela |

### 3.4. Pondération

La plupart du temps, si la note est de 1 ou 2 à la première ligne, les autres lignes deviennent non applicables et ne doivent pas être notées. Dans ces situations, la note finale est donc la note obtenue à cette première ligne.

- **ITEM 4: ANALYSE**

**OBJECTIF: Analyser la performance des apprenants pour identifier leur(s) lacune(s) : effectuer une bonne analyse des actions des apprenants en contexte (« contextualisation », dans la situation vécue)**

### 4.1. Capacité de discuter de la performance des apprenants

| NOTATION | 1 | 2 | 3 | 4 | 5 |
|---|---|---|---|---|---|
| **INDICE(S)** | **Ne discute pas** de la **performance** des apprenants dans le contexte du cas vécu | N'encourage **pas** de discussion de la **performance** des apprenants dans le contexte du cas vécu | Encourage une discussion de la **performance** des apprenants dans le contexte du cas vécu | Encourage une discussion de la **performance** des apprenants dans le contexte du cas vécu | Encourage une discussion de la **performance** des apprenants dans le contexte du cas vécu |
| **EXEMPLES** | - Le D omet complètement et/ou délibérément de discuter de la performance des apprenants pendant la simulation<br>- Le D effectue un cours magistral à la place d'un débreffage | Le D discute partiellement de la performance des apprenants dans le contexte de la simulation | - Le D discute entièrement de la situation vécue avec les apprenants<br>- Le D encourage les apprenants à expliquer ce qu'il s'est passé pendant la situation simulée à travers leurs propres yeux<br>- Le D encourage les apprenants à verbaliser leurs sentiments et le cadre de référence qui a sous-tendu leur action | | |

### 4.2. Exploration des cadres de référence ayant sous-tendu les actions des apprenants

| NOTATION | 1 | 2 | 3 | 4 | 5 |
|---|---|---|---|---|---|
| **INDICE(S)** | N'explore **pas** les **processus cognitifs sous-jacents** à la performance des apprenants | N'explore **pas** les **processus cognitifs sous-jacents** à la performance des apprenants | N'explore **pas** les **processus cognitifs sous-jacents** à la performance des apprenants | Explore les **processus cognitifs sous-jacents** à la performance des apprenants | Explore **efficacement** les **processus cognitifs sous-jacents** à la performance des apprenants |
| **EXEMPLES** | - Le D omet ou évite d'explorer les cadres de référence des apprenants<br>- Exposition passive des cadres de références par les apprenants, non enrichie par le D<br>- Absence de confrontation du cadre de référence du D avec celui ou ceux des apprenants | | | - Exploration incomplète ou inadéquate des cadres de référence des apprenants (avec questions socratiques ou empreintes de jugement)<br>- Confrontation des cadres de référence du D avec celui ou ceux des apprenants | - Exploration complète, avec un bon jugement, des cadres de référence des apprenants<br>- Confrontation des cadres de référence du D avec celui ou ceux des apprenants |

## • ITEM 5: TRANSFERT

**OBJECTIF: Combler les lacunes des apprenants (décontextualisation), et encourager le transfert des apprentissages (recontextualisation)**

### 5.1. Capacité de mettre en évidence les principes généraux (abstraits) découlant de la situation simulée comblant les lacunes des apprenants (décontextualisation)

| NOTATION | 1 | 2 | 3 | 4 | 5 |
|---|---|---|---|---|---|
| **INDICE(S)** | Fait émerger des **principes généraux erronés** compte tenu de la situation vécue | **Ne fait pas** émerger les **principes généraux** découlant de la situation vécue (connaissances et/ou stratégies de prise en charge) | **Essaie de faire** émerger les **principes généraux** découlant de la situation vécue (connaissances et/ou stratégies de prise en charge) | Fait émerger les **principes généraux** découlant de la situation vécue (connaissances et/ou stratégies de prise en charge) | Fait émerger les **principes généraux** découlant de la situation vécue (connaissances et/ou stratégies de prise en charge) |
| **EXEMPLES** | - Principes généraux erronés<br>- Principes généraux dépassés (non à jour) | - Aucune mention des principes généraux découlant de la situation simulée pendant le débreffage | - Principes généraux partiellement adaptés à la performance des apprenants<br>- Le D permet aux apprenants de partiellement combler leurs lacunes | - Principes généraux parfaitement adaptés à la performance des apprenants<br>- Le D permet aux apprenants de combler toutes leurs lacunes ou presque<br>- Les apprenants ont acquis les principes généraux qui pourront être mobilisé dans d'autres situations similaires réelles ou simulées | |

### 5.2. Capacité de proposer des stratégies concrètes découlant de la situation simulée comblant les lacunes des apprenants (décontextualisation)

| NOTATION | 1 | 2 | 3 | 4 | 5 |
|---|---|---|---|---|---|
| **INDICE(S)** | Ne débreffe **que** sur des points purement **abstraits** | Débreffe plutôt sur des points purement **abstraits** | Débreffe plutôt sur des points purement **abstraits** | Provoque **l'émergence de stratégies concrètes de prise en charge** (pas de débreffage sur des points abstraits) | Provoque **l'émergence de stratégies concrètes de prise en charge** (pas de débreffage sur des points abstraits) |
| **EXEMPLES** | Le D consacre tout le temps du débreffage à un cours magistral sans faire émerger de stratégie concrète | - Le D consacre une partie du temps du débreffage à des rappels de cours<br>- Pas ou peu de stratégies concrètes proposées, et/ou inadaptées à la situation vécue | | - Le D facilite l'émergence de stratégies concrètes découlant de la situation vécue et appropriées à la gestion d'un cas similaire en pratique clinique | |

### 5.3. Capacité de projeter les apprenants dans une nouvelle situation (recontextualisation)

| NOTATION | 1 | 2 | 3 | 4 | 5 |
|---|---|---|---|---|---|
| **INDICE(S)** | **Ne replace pas** l'apprenant en situation nouvelle (prototypique, similaire) | **Ne replace pas** l'apprenant en situation nouvelle (prototypique, similaire) | **Ne replace pas** l'apprenant en situation nouvelle (prototypique, similaire) | **Ne replace pas** l'apprenant en situation nouvelle (prototypique, similaire) | **Replace** l'apprenant en situation nouvelle (prototypique, similaire) |
| **EXEMPLES** | - Le D ne projette pas les apprenants dans une nouvelle situation pendant le débreffage<br>- Le D n'explicite pas les aspects prototypiques du cas, ou ne questionne pas les apprenants à ce sujet | | | | Le D explicite les aspects prototypiques de la situation, ou questionne les apprenants à ce sujet, puis les guide dans l'application de ces concepts dans une nouvelle situation similaire |

**5.4. Pondération :** si la note est différente pour chaque ligne, alors la présence d'une phase de recontextualisation (3e ligne) pèse plus dans la note finale de l'item que l'émergence de stratégies concrètes (2e ligne) ou la discussion des principes généraux relatifs à la situation vécue (1e ligne).
